# Deciphering HER2-HER3 Dimerization at the Single CTC Level: A Microfluidic Approach

**DOI:** 10.3390/cancers14081890

**Published:** 2022-04-08

**Authors:** Ezgi Tulukcuoglu Guneri, Emile Lakis, Ismail Hajji, Elian Martin, Jerome Champ, Aurore Rampanou, Jean-Yves Pierga, Jean-Louis Viovy, Charlotte Proudhon, François-Clément Bidard, Stéphanie Descroix

**Affiliations:** 1 Laboratoire Physico Chimie Curie, CNRS UMR168, Institut Curie, 75005 Paris, France; ezgitulukcuoglu@yahoo.com (E.T.G.); emile.lakis@gmail.com (E.L.); ismail.hajji@curie.fr (I.H.); martin.elian@free.fr (E.M.); jerome.champ@gmail.com (J.C.); jean-louis.viovy@curie.fr (J.-L.V.); 2 PSL Research University, Sorbonne Université, 75005 Paris, France; 3 Circulating Tumor Biomarkers Laboratory, SiRIC, Translational Research Department, Institut Curie, PSL Research University, 75005 Paris, France; aurore.ramanou@curie.fr (A.R.); jean-yves.pierga@curie.fr (J.-Y.P.); charlotte.proudhon@curie.fr (C.P.); francois-clement.bidard@curie.fr (F.-C.B.); 4 INSERM U934 CNRS UMR3215, Institut Curie, PSL Research University, 75005 Paris, France

**Keywords:** circulating tumor cells, proximity ligation assay, HER2, HER3 dimerization, microfluidic

## Abstract

**Simple Summary:**

Among these different biomarkers, circulating tumor cells have proven to be of high interest for different types of cancer and in particular for breast cancer. Here we focus our attention on a breast cancer subtype referred as HER2-positive breast cancer, this cancer being associated with an amplification of HER2 protein at the plasma membrane of cancer cells. Combined with therapies targeting the HER2 protein, HER2-HER3 dimerization blockade further improves a patient’s outcome. In this work, we propose a new approach to CTC characterization by on-chip integrating proximity ligation assay, so that we can quantify the HER2-HER3 dimerization event at the level of single CTC.

**Abstract:**

Microfluidics has provided clinicians with new technologies to detect and analyze circulating tumor biomarkers in order to further improve their understanding of disease mechanism, as well as to improve patient management. Among these different biomarkers, circulating tumor cells have proven to be of high interest for different types of cancer and in particular for breast cancer. Here we focus our attention on a breast cancer subtype referred as HER2-positive breast cancer, this cancer being associated with an amplification of HER2 protein at the plasma membrane of cancer cells. Combined with therapies targeting the HER2 protein, HER2-HER3 dimerization blockade further improves a patient’s outcome. In this work, we propose a new approach to CTC characterization by on-chip integrating proximity ligation assay, so that we can quantify the HER2-HER3 dimerization event at the level of single CTC. To achieve this, we developed a microfluidic approach combining both CTC capture, identification and HER2-HER3 status quantification by Proximity Ligation Assay (PLA). We first optimized and demonstrated the potential of the on-chip quantification of HER2-HER3 dimerization using cancer cell lines with various levels of HER2 overexpression and validated its clinical potential with a patient’s sample treated or not with HER2-targeted therapy.

## 1. Introduction

Breast cancer is the most frequent cancer in women and is composed of distinct molecular subtypes associated with different clinical outcomes [1]. About 15–20% of all breast cancer cases display an amplification and/or overexpression of the *ERBB2* gene, which encodes the human epidermal growth factor receptor 2 (HER2), and are referred to as “HER2-positive breast cancer” [2,3]. HER2 is a transmembrane receptor, member of the human epidermal growth factor family, along with HER1/EGFR, HER3 and HER4. In HER2-positive breast cancer, the *ERBB2* amplification leads to an overexpression of the HER2 protein at the cell plasma membrane, this large amount of HER2 increases the activation of downstream proliferating signaling pathways by HER2 homo- or heterodimers [2].

In the clinics, a first breakthrough was achieved by the anti-HER2 monoclonal antibody trastuzumab, which inhibits ligand-independent HER2/HER3 signaling and triggers an immunogenic response, such as antibody-dependent cell-mediated cytotoxicity [4]. The use of this targeted therapy was associated with an impressive improvement of clinical outcomes for HER2-positive breast cancer patients, including major extension of the life expectancy and lower relapse rates [5]. A second humanized monoclonal antibody that binds to a distinct epitope of HER2, pertuzumab, was then investigated in combination with trastuzumab to further increase the blockade of HER2-signaling. Pertuzumab specifically inhibits HER2-HER3 dimerization at the cell plasma membrane and its downstream signaling. The addition of pertuzumab leads to significant gains in overall survival as compared to trastuzumab alone, both in metastatic and nonmetastatic HER2-positive breast cancer patients [6]. However, subgroup analyses on pathological and clinical characteristics have not retrieved any specific biomarker that could predict the benefit of pertuzumab, in order to identify which patient to treat with this combination [7]. So far, the main biomarker to be eligible for this type of targeted therapy relies on the overexpression of HER2 in the primary tumor.

Recently, assessing HER2-HER3 dimerization level on tumor tissue, however, has demonstrated its potential for being used as a predictive biomarker. In particular, determining the HER2-HER3 dimerization status of FFPE tumor tissue using FRET-FLIM (Förster resonance energy transfer-Fluorescence Lifetime Imaging Microscopy) has been shown to predict the likelihood of metastatic relapse up to 10 years after surgery independently of tumor HER2 expression [8]. Chromogenic Proximity Ligation Assay has also been used to assess the HER2-HER3 dimerization status in early primary breast cancer series, submitted or not to trastuzumab treatment. The authors showed that these dimers were detected in 13% of breast cancers in general and in 73% of HER2+ tumors, and that the loss of p21 expression mediated through HER2-HER3 heterodimerization is associated with poor outcome for trastuzumab-treated HER2+ patients [9]. These studies provoke interest in assessing the status of HER dimerization on tumor cells for better patient stratification. This, however, has so far been limited to investigation performed on solid tumor tissues only.

It is now well established that tumor material is also accessible from the blood stream and that circulating biomarkers are a major asset for cancer diagnosis and monitoring [10,11]. Among the different types of circulating biomarkers [12,13], circulating tumor cells (CTC) have been demonstrated as a prognosis factor for different cancer types and in particular for breast cancer [14,15]. They have also proven to reflect the treatment efficacy, demonstrating the dynamic character of this biomarker [16]. CTC are malignant cells released from primary or secondary tumor sites in the blood stream, with the potential to disseminate to distant organs and give rise to metastases. HER2-HER3 dimerization status has not been evaluated on CTCs yet. Investigating the HER2-HER3 dimerization status from CTCs might be relevant as a potential non-invasive predictive marker of pertuzumab efficacy.

Here, we report the first microfluidic approach to quantify HER2-HER3 dimerization at single CTC level using a Proximity Ligation Assay (PLA) performed on-chip. To achieve this, we developed a microfluidic approach that combines both CTC capture and HER2-HER3 status identification, in a quantitative manner. We first optimized and demonstrated the potential of the on-chip quantification of HER2-HER3 dimerization at the single-cell level using cancer cell lines, and further demonstrated its implementation on blood samples from breast cancer patients treated or not with HER2-targeted therapy.

## 2. Materials and Methods 

### 2.1. Microfluidic Device Fabrication

Top part. The principle of the Ephesia system used for CTC capture and the channels design are described in [17]. The channels were replicated on a 5 cm diameter cyclo-olefin copolymer (COC) substrate (5 mm thickness Topas^®^, 8007) using a nickel master (custom made by AREMAC-Polymer, Lyon France). COC substrates were embossed by using a SQUAMEX 3T (set at 50 °C) with a pressure of 3 mbar for 25 min at 100 °C, then cooled down to 50 °C. To connect liquid injection tubing (PTFE tubing with 0.56 mm ID × 1.07 mm OD, Bohlender, Grunsfeld, Germany), two holes were made both in inlet and outlet using 1.1 drill on the top part of the chip.

Bottom part. It was replicated from a NOA master mold (Norland Optical Adhesive 81) onto a 245 µm COC substrate (Topas^®^, 8007) using a dry film laminator (RLM 419p) at 140 °C with a speed of 0.2 mm/min and a load of 4.5 kg pressure. NOA master mold was fabricated from sacrificial PDMS replica (Dow Corning Sylgard 184 prepared with a 10:1 ratio of based and curing agents respectively), made in soft lithography as described in [18] by pouring NOA onto a flexible copper sheet and exposing to a UV lamp for 10 min. Thereafter, carboxyl acid group functionalized magnetic beads (Dynabeads^®^ M-270 Carboxylic Acid, ThermoFisher Scientific, Paris, France) were capillary assembled into the micropatterned holes on the bottom part by trapping the particles into recessed structures and dragging a droplet of particle suspension under confinement using a motorized stage with a speed of 25 µm/second at room temperature [19,20]. Particle suspension consisted of a solution of 150 μL beads resuspended in 1 mL of distilled water containing 10 μL of Triton X45 (0.1%) and SDS (0.01 M) at 1:1 ratio.

Bonding. The up and bottom part was sealed using 15% hexadecane/isopropanol (*v*/*v*) solution. The upper part was put in contact with lintless paper impregnated with hexadecane/isopropanol, and then the two parts were aligned. The assembly was achieved by using hot press with 3 mbar pressure for 15 min at 75 °C with the help of PDMS (polydimethylsiloxane) substrate placed on the top part of the chip and facing the bottom part to the hot plate.

### 2.2. Temperature Control in the System

A specific feature of the Ephesia system is the need to keep the magnetic field on during the entire experimental manipulation for the magnetic columns to remain intact, necessary for imaging analysis. Therefore, heating was supplied with a benchtop heating plate (Stuart, SD160) in which the microscope stage was mounted with the magnetic coil around the microchip. The temperature was controlled by a thermocouple placed in the center of the chip. The calibration of the temperature (37 °C) was necessary in the chip due to heat loss by dissipation to microscope stage and the surroundings. Moreover, the minimum time to reach the desired temperature was optimized.

### 2.3. Cell Culture

Breast cancer cell lines MCF7, MDA-MB-231 and SK-BR-3 were purchased from ATCC and cultured at 37 °C in a humidified atmosphere with 5% CO_2_ using high-glucose GlutaMAX™ DMEM (MCF-7, MDA-MB-231) and McCoy’s 5A (Modified) Medium (SK-BR-3) supplemented with 10% (*v*/*v*) FBS (fetal bovine serum), 1% (*v*/*v*) penicillin–streptomycin. Cell lines were characterized by flow cytometry and immunofluorescence for HER2 and HER3 expression (Figure S1). For microchip experiments, 10 k cells of each cell line were injected inside Ephesia.

### 2.4. Gene Knockdown

For siRNA transfections, DharmaFECT1 (Dharmacon, Horizon, Cambridge, UK) was added to 10 nM siHER2/HER3 ON-Targetplus smartpool (Dharmacon, Horizon). siGAPDH was used a transfection positive control and siNon-targeting as a negative control. After optimization, we set 72 h of incubation; cells were then collected for injection inside Ephesia or harvested for RNA extraction. mRNA HER2 and HER3 were assessed by (RT-qPCR).

### 2.5. RNA Extractions and Reverse Transcription

RNA was extracted from SK-BR-3 cell line using RNeasy Mini Kit (Qiagen, Les Ulis, France). One microgram total RNA was reverse-transcribed using High-Capacity cDNA Reverse Transcription Kit (Applied Biosystems, San Francisco, CA, USA).

### 2.6. Real Time-Quantitative PCR

cDNA amplification was performed using KiCqStart^®^ SYBR^®^ Green qPCR ReadyMix™ (Merck, Darmstadt, Germany). A reaction cocktail without the sample template (cDNA) was prepared to reduce pipetting errors and maximize assay precision. First, 15 μL of the reaction cocktail with all the required components except the sample template (cDNA) were dispensed into separate SmartCycler^®^ reaction tubes (Cepheid Inc., Sunnyvale, NS, Canada). Then, 5 μL of the sample template (cDNA) were added to each reaction as the final step. All real-time PCR reactions were performed in triplicates using a SmartCycler^®^ automated real-time PCR system (Cepheid Inc., Sunnyvale, NS, Canada). See primers and thermal conditions in Tables S1 and S2.

### 2.7. Patient Recruitment and Blood Sample Processing

Blood samples were obtained from patients with HER2-negative and HER2-positive metastatic breast cancer treated at Institut Curie (Paris, France). These samples were obtained as part of an ethically approved study on circulating biomarkers (ALCINA, NCT02866149), to which patients gave written informed consent.

To prepare the patient sample, RosetteSep cocktail is mixed with blood sample (10 mL) and incubated for 20 min at room temperature. The mixture is then diluted by PBS-BSA2% at a ratio of 1:1 and layered over density gradient medium (Ficoll-Paque PLUS) and centrifuged at 1200× *g* for 30 min at 20 °C without breaks. Retrieved peripheral blood mononuclear cells (PBMCs) are diluted with 300 µL PBS-BSA 2% and centrifuged at 90× *g* for 10 min without breaks. Lastly, supernatant is removed, and the cell pellet is resuspended in 200 μL of PBS-BSA 1% solution.

### 2.8. In-Situ Indirect and Direct Proximity Ligation Assay on Glass Slides

Direct PLA protocol was performed as recommended by manufacturer’s instructions using Duolink^®^ Orange in-Situ detection reagents and PLA probes. Cells were seeded on an 8-well glass slide (Millicell EZSLIDE 8-well glass, sterile, Merck Millipore). After 24 h of cultivation, the cells were fixed with 4% PFA for 20 min and permeabilized with 0.1% Triton 100-X (*v*/*v*, Molecular Probes™) for 20 min. After stringent washing with solution A for 10 min, cells were incubated with blocking solution for 30 min at 37 °C and followed by: 

For direct PLA (as recommended by Duolink supplier), primary antibodies were incubated overnight at 4 °C with 1000-fold dilution. After stringent washing, PLA probes were incubated at 37 °C for 1 h. PLA probes were chosen according to species of primary antibodies, which are different form each other. Later, ligation and RCA were performed as described below. Each incubation step was followed by washing with PBS-BSA 1%.

For indirect PLA: Without any washing step, PLA probes were incubated at 4 °C overnight. Thereafter, ligation at 37 °C for 30 min and RCA (Rolling Circle Amplification) reaction at 37 °C for 100 min were performed followed by washing steps, respectively, with solution (sol) A and B. Finally, the glass slide was dried and mounted by DAPI-mounting media. PLA probes were created by conjugating oligonucleotides directly on the primary antibodies using Duolink ProbeMaker kit according to user guide instructions using the following primary antibodies: HER-2 (1:100/Thermo Scientific™, clone e2–4001), HER-3 (1:100/Thermo Scientific™, clone 2F12). During incubation times, 8-chamber hybridization gaskets (Secure-Seal™, ThermoFisher) were used to avoid drying the solutions for each PLA method.

### 2.9. In-Situ Proximity Ligation Assay in Ephesia Microfluidic Device

To implement PLA into our system, various optimizations were performed using control cell lines, which led to the following protocol. First, 10,000 cells were injected into the microchip using MFCS-8C flow controller (Fluigent, Le Kremlin-Bicêtre, France) with a flow rate of ~30 µL/min. Once cells were captured on anti-EpCAM conjugated magnetic beads (Dynabeads^®^ Epithelial Enrich), they were fixed with 4% PFA for 20 min and permeabilized with 0.1% (*v*/*v*) Triton X-100 for 20 min. Two blocking steps were applied with a solution of salmon sperm DNA (PBS-BSA 1%, Tween 0.05% with 100 µM salmon sperm) and provided by ready-to-use solution at 37 °C for 30 min, then at room temperature overnight. PLA probes were incubated for 1 h at 37 °C and followed by a washing step with sol A at RT (1 mL, ~30 min). A ligation step was performed for 30 min at 37 °C, followed by the washing step (sol A, 500 µL, 15 min). An RCA step was performed for 100 min at 37 °C, followed by a washing step with sol B (2 mL, ~1 h). Cells were then stained with anti-cytokeratin (4.7 mg/mL, Dako, M3515, clone AE1/AE3) labeled with Zenon Alexa 488 (57.6 µg antibody/1 µL labeling reagent) and CD45 Monoclonal Antibody (MEM-28), Alexa Fluor 647 (1:25) for 30 min followed by washing with 1% BSA in PBS and 0.01% sol B. Finally, DAPI-mounting media (1:2 in water, *v*/*v*) was injected. Imaging was performed at least 15 min after DAPI injection.

### 2.10. Imaging and Signal Quantification

Images were captured by inverted epifluorescence microscope (NIKON TI-E) equipped with Photometrics CoolSNAP HQ2 CCD camera and an automatized stage for both chip (CFI Plan Apo VC 60XWI, NIKON) and glass slide (CFI Plan Fluor 40X). Image acquisition was performed by using NIKON software. Z-stack images were first treated with Fiji, using background subtraction, gamma and maximum intensity projection functions. Then, PLA signals were quantified using CellProfiler (2.2.0, rev ac0529e) in the following modules: Identify primary objects/identify objects manually for defining nuclei using DAPI staining; identify secondary object for defining cytoplasm using cytokeratin (CK) staining or DAPI staining with N-distance method; enhance speckles features to enhance PLA signals and then identify primary objects for defining PLA signals. For each cell, the presence of cytokeratin staining was determined using the thresholding based on fluorescence signal intensity on the background. Object enhancing and filtering size were adjusted depending on the objective used for control experiments on chip and on glass slides, which also varied between patient samples.

## 3. Results

### 3.1. HER2-HER3 PLA Optimization on Coverslip

**Figure 1:**
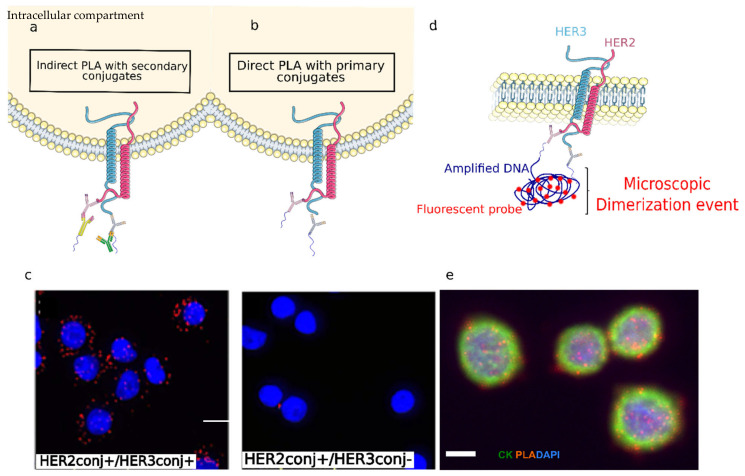
(**a**) Schematic representation of indirect PLA; primary antibodies are detected by PLA conjugated secondary antibodies. (**b**) Direct PLA depicted by conjugated primary antibodies only. (**c**) Direct PLA signals detected in SK-BR-3 cells in the presence of HER2 and HER3 antibodies (left). No signals detected after omitting HER3 antibody (right). (**d**) Schematic representation of the direct PLA-Assay. (**e**) PLA coupled with immunostaining with SK-BR-3 cells on glass slides. Scale bar is 20 µm (**c**,**d**), 10 µm (**e**). Schemes were created by using Servier Medical Art, 2021.

To assess the HER2-HER3 dimerization status of single CTCs, we used the Proximity Ligation Assay (PLA), which has already demonstrated its potential in tumor. PLA is an in-situ immuno-based technique used to study protein-protein interactions, location and quantification at the single molecule level [21,22]. It consists of targeting interacting proteins of interest with two specific primary antibodies. The bound targets are then detected by a pair of secondary antibodies coupled to oligonucleotides probes. When the probes are in close enough proximity (less than 40 nm), the two ends of the probes can be ligated efficiently in presence of hybridizing connector oligos, resulting in a circular single-strand DNA. This DNA is then used as template for isothermal amplification by rolling circle amplification (RCA), in the presence of a primer complementary to a sequence of this circular DNA and of a polymerase with strand-displacement activity. Amplified DNA circles are further detected by hybridization of complementary fluorescent probes, which highlight each molecular dimerization event as a micrometric fluorescent single dot. The most widely used approach for PLA is the indirect PLA described above, where secondary antibodies coupled to oligonucleotides probes are used. The secondary antibodies, called PLA probes, are directed against the constant regions of the primary antibodies which are raised in different species (Figure 1a).

Although highly informative, PLA is a meticulous and tedious process, which includes several steps involving incubation of multiple reagents, heating conditions combined with enzymatic reaction, as well as a DNA amplification and hybridization phase. We first optimized the PLA conditions with EpCAM-positive cancer cell lines, which model the epithelial phenotype of CTCs, seeded on glass coverslips. In particular, we first optimized the cell permeabilization time and antibodies concentrations. We used cells lines, with known high and low HER2 expression levels: HER2-amplified SKBR-3 (score 3+) and HER2-low MDA-MB-231 (score 0–1+) [23]. Their HER2 expression level has been confirmed by FACS.

As expected, we observed more HER2-HER3 PLA signals per cell for SK-BR-3 than MDA-MB 231 cell line (Figure S1), however, before integrating PLA on chip, we evaluated the assay specificity by omitting one of the primary antibodies or both. Unexpectedly, we observed a large number of PLA signals in SK-BR-3 cells even in the absence of primary antibodies highlighting the presence of non-specific interactions of the PLA probes (Figure S2). This low specificity of the assay is critical in these conditions as the positive signal achieved with low HER2 expression (MDA-MB 231) cell line is of the same order of magnitude or even lower than the negative and nonspecific signal obtained with SK-BR-3.

To improve the assay specificity, different protocol modifications have been considered, in particular, we decreased the primary antibody concentration to limit the nonspecific adsorption and modified the probe combination. However, none of these optimizations has permitted to avoid nonspecific signals. To improve the assay specificity, we finally consider direct PLA that employs primary anti-HER2 and anti-HER3 antibodies directly conjugated to oligonucleotide probes (Figure 1b–d). Test experiments performed on glass coverslips demonstrated that direct PLA is specific and no signal is observed when omitting the anti-HER3 antibody (Figure 1c).

Another essential aspect of the analysis procedure was to combine PLA with immunostaining of several markers needed to assess with certainty tumoral status of each captured cell. CTC are defined as epithelial nucleated cells (DAPI+, cytokeratin or CK+) staining negatively for C45 (CD45−); thanks to immunostaining, they can be distinguished from contaminating endothelial cells that are often found in CTC analysis due to the imperfect selectivity of the capture process. We showed that immunostaining could not be performed prior to PLA, as signals are affected by PLA conditions, in particular, heating steps. However, we were able to perform HER2-HER3 PLA followed by immunostaining. In these conditions, we can successfully detect both the number of HER2-HER3 dimerization events and the CTCs markers: DAPI+ CK+ CD45 (Figure 1e).

### 3.2. HER2-HER3 Dimerization of Single CTC: On-Chip PLA Integration

**Figure 2:**
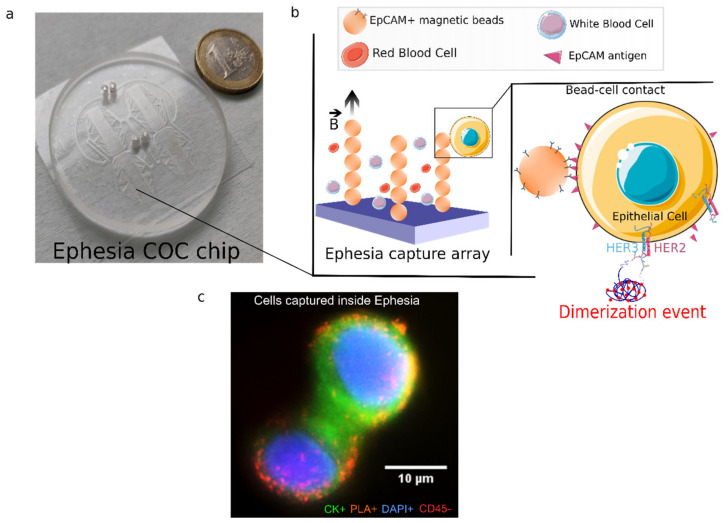
(**a**) Picture of the COC Ephesia chip. (**b**) Schematic representation of Ephesia CTC immunoextraction; captured cells in the array of self-assembled anti-EpCAM beads are subjected to on-chip PLA. B arrow shows the magnetic field direction. (**c**) SK-BR-3 cells captured inside Ephesia device showing PLA signals (orange) and stained for cytokeratin CK (green), CD45 (red) and DAPI (blue). Scale bar is 10 µm. (Schemes were created by using Servier Medical Art, 2021.

Investigating on-chip HER2-HER3 dimerization of single CTCs first requires an efficient capture of CTCs from patient samples. We chose the Ephesia microfluidic approach, which selectively captures CTCs [16]. The Ephesia device relies on immunoextraction of CTCs (Figure 2a,b). Briefly, the Ephesia system relies on immunomagnetic enrichment, whereby cells are captured on an array of self-assembled magnetic pillars formed by superparamagnetic beads functionalized with anti-EpCAM antibodies. Magnetic columns are created using an electromagnetic coil generating a magnetic field of 30 mT. The top part of the chip comprises the four capture chambers with a total size of 3.3 cm in length and 3 mm in width, with optimized diamond shape flow channels. The bottom part of the chip is patterned with 48,000 holes in a hexagonal array of 48 rows and 1000 lines with 60 μm spacing and a 15 μm diameter of circles with 10 μm depth. This pattern determines the design of the magnetic pillars and is designed to provide the optimum cell capture efficiency. This device demonstrates improved performances as compared to the Cellsearch (Veridex J&J) approach with about 70% of capture efficiency of CTCs from blood samples [16]. Implementing on-chip PLA to analyze single CTCs required extensive redesign of the Ephesia workflow. First, to make the device compatible with PLA experimental conditions, we used cyclic olefin copolymer (COC) as bulk material instead of polydimethylsiloxane (PDMS), which was previously used. Its gas impermeability, combined with its excellent optical properties, makes COC an optimal material to combine on-chip CTC capture and PLA. A new microfabrication protocol was thus developed to produce COC Ephesia chips (Figure 2a). In particular, it includes hot embossing using nickel master produced by LIGA process on a stainless-steel disc for the top part that contains channels below 100 µm in width. The bottom part of the chip was replicated from a NOA master mold onto a thin COC substrate (thickness: 245 µm) by roll-embossing. Finally, we optimized the chip bonding using a solvent-assisted bonding approach (see Mat Met) [24].

**Figure 3:**
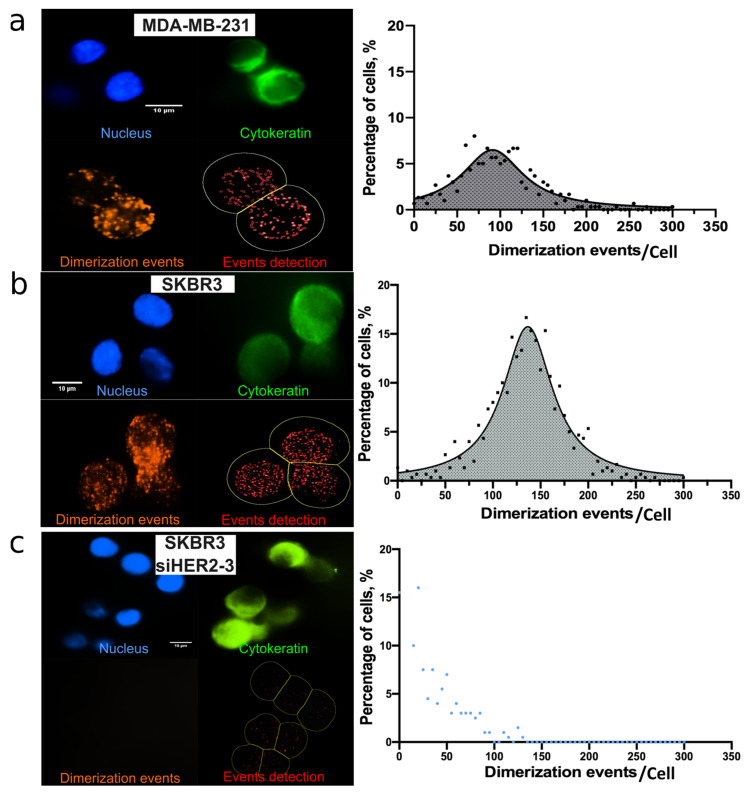
Left panel: On-chip immunofluorescence images of (**a**) MDA-MB-231 cells and (**b**) SK-BR-3 cells captured inside Ephesia showing PLA signals (left panel, orange). (**c**) HER2/HER3 gene silencing in SK-BR-3 cells induced a decreased in PLA signals, represented by the blue Gaussian curve shifted to the left. Right panel: Graphs quantifying the dimerization events per cell (bottom right, red) in each condition. *n* = 3 independent experiments for each condition. Scale bar: 10 µm.

Further experiments were necessary to implement the HER2-HER3 PLA on-chip. Indeed, the presence of magnetic beads, the increased surface-to-volume ratio, as well as the use of hydrophobic COC chips drastically affect PLA workflow and performances. In particular, the antibody concentrations, the washing and blocking steps have been modified to achieve high-quality signals on cells captured in the Ephesia chip. The optimal conditions include the dilution of primary antibodies at 1/100 ratio, a blocking step overnight at room temperature and longer washes (see details in Section 2). This optimized protocol was validated with SK-BR-3, as illustrated in Figure 2c, and MDA-MB-231 cell lines captured in the Ephesia chip (Figure S3). We observed distinct PLA dots scattered all over the cells for both SK-BR-3 and MDA-MB-231. This was the first demonstration of on-chip HER2-HER3 proximity ligation assay implementation. PLA signal quantification was next established using Fiji and Cell Profiler, as detailed in Section 2. A theoretical cell membrane was generated (yellow) in order to assign each PLA dot (red) to the corresponding cell; this allowed to quantify the number of PLA signals per cell (Figure 3). As expected, we observed wide distributions of the number of HER2-HER3 dimerization events per cell in the two cell lines, independently of their HER2 expression level. The experimental spreading of data could be fitted by Gaussian distributions, and we observed average values of 132 ± 40 PLA signal per cell for SKBR3 cells and 94 ± 44 for MDA-MB-231 cells (Figure S4). These results were consistent with our observations using Fluorescence-Activated Cell Sorting (FACS), which showed a large distribution of HER2 expression.

To further demonstrate the specificity of the PLA signals achieved on chip, we knocked down the HER2 and/or HER3 genes in SK-BR-3 cell line, using siRNA. We performed gene silencing of HER2 and HER3 individually, as well as in combination (Figure S5). We observed a strong decrease of the number of PLA signals per cell in cells treated with siHER2 or siHER3 (Figure S6) and even more when both HER2 and HER3 gene were knocked down (Figure 3c). We observed a drop from an average value of 132 PLA signals/cell, in the proficient cells, to 24 PLA signals/cell in HER2/HER3 knocked-down cells (Figure 3c). Altogether, these results demonstrate the specificity of the CTC-assay and confirm that the epithelial status of cells epithelial cell captured in the Ephesia chip can be combined with PLA to assess the HER2-HER3 dimerization events at the single-cell level in a quantitative and specific manner.

### 3.3. On-Chip Single CTC PLA Analysis from Metastatic Breast Cancer Patients’ Blood

In order to demonstrate the clinical potential of this assay, we performed the same analysis on blood samples from metastatic breast cancer (MBC) patients. A flowchart depicting patients’ recruitment is detailed in Figure S7. A total number of 43 MBC patients was included. Blood samples were available for *n* = 38 patients, and missing for *n* = 5 patients. Twenty-three and fifteen patients were treated for HER2+ and HER2− metastatic breast cancer, respectively (HER2 status being assessed on the last tumor tissue available). In HER2- MBC, six (26%) patients showed a detectable level of CTCs in their blood via Ephesia immuno-extraction, while three (20%) HER2+ patients had CTCs detected in their blood. In parallel, CTC detection was first assessed for each sample using the gold standard method CellSearch before proceeding with the on-chip assay.

**Figure 4:**
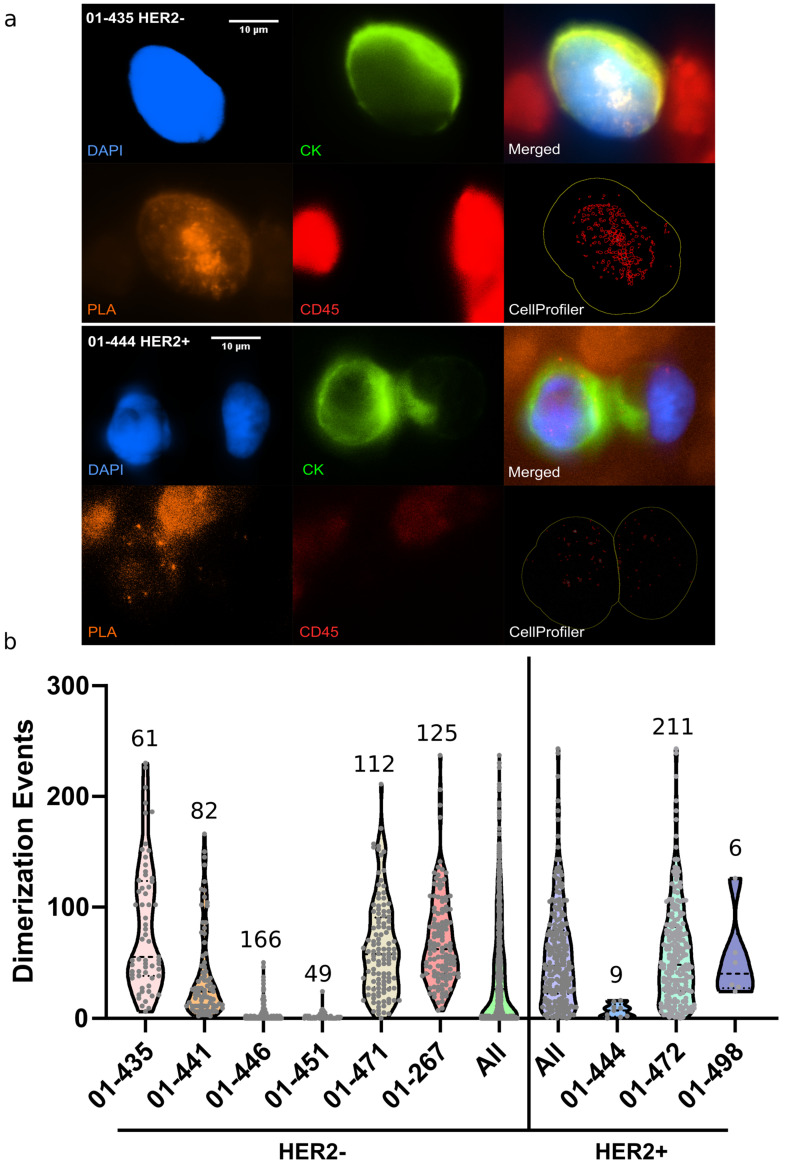
(**a**) Examples of CTCs detected from patient blood with MBC HER2− or HER2+, using the Ephesia chip. CTCs were further subjected to PLA and immunostaining for CK, DAPI and CD45 detection. (**b**) Violin plots showing the distribution of HER2-HER3 PLA signal per cell (number of CTC detected indicated for each patient sample). Scale bar is 10 µm.

Similarly to cell line experiments, sequential CTC capture, HER2-HER3 PLA followed by immunostaining for CTC identification (DAPI+, CK+, CD45−) were performed on chip for each patient sample. Figure 4a presents examples performed on blood from HER2− (01-435) or HER2+ (01-444) patients. We next characterized the distribution of PLA signal per CTC for each patient with detected CTCs (Figure 4b). We observed a wide heterogeneity of the HER2-HER3 PLA profiles (Figure 4b). About one-third of the patient’s samples present a low average value of PLA signal per CTC along with a narrow distribution whereas the other samples present a large distribution of number of HER2-HER3 dimerization events/per CTC. It is worth noting that both the average value of PLA signal per CTC and its distribution do not correlate with the number of CTC in the patient sample. These parameters are also independent of the primary tumor HER2 status (Figure 4b). This potentially reflects the heterogeneity of CTC HER2 status independently of the primary tumor HER2 status [25]. Finally, the profile diversity could also be linked to the treatments received by the patients (chemotherapy, trastuzumab, pertuzumab, T-DM1, etc.). These patient samples were mostly analyzed to validate the on chip HER2/3 PLA for single CTC, however, regarding the interest of the biomarker itself, the small number of patients analyzed at stage and that received a similar treatment does not allow us to perform further correlation analysis.

Finally, these data demonstrate the possibility to assess the HER2-HER3 status of single CTCs directly from blood samples by combining on-chip CTC capture, identification and PLA. However, in the future, a larger number of samples should be analyzed to further assess how this status could be correlated with patient response to treatment.

## 4. Conclusions

This study reports the first quantification of HER2-HER3 dimerization events at the single circulating tumor cell level. This analysis is based on the combination of microfluidic sorting of CTCs by the Ephesia technology, with on-chip PLA. PLA is usually performed on fixed cell lines on a glass substrate or on FFPE tissues [7,26,27]. Here, a new generation of Ephesia device was first developed and optimized to make it compatible with on-chip PLA. Then, we adapted the PLA experimental conditions to be performed within the chip. In particular, we optimized the permeabilization time, antibody concentrations and immunostaining of CTCs to improve the signal quality on chip and render the PLA compatible with simultaneous CTC identification. To improve the assay specificity, a direct PLA format was chosen for further experiments to avoid the nonspecific signal observed with an indirect PLA method. Two breast cancer cell lines were used to validate on-chip PLA conditions: SK-BR-3 and MDA-MB-231 with a high and low HER2 expression profile, respectively. The wide distribution of the number of PLA signal/cell detected is consistent with their HER2 expression levels. The specificity of the signals was further evidenced by knocking down HER2 and/or HER3 using siRNA. Finally, we demonstrated the possibility of using our optimized method for clinical samples analysis. Both HER2− and HER2+ metastatic breast cancer patients were analyzed. Our results show a wide diversity of mean values and distributions of the PLA signal/CTC among patients with no clear correlation with the total number of CTC detected, nor with the HER2 status of the patient’s primary tumor. After this first proof of concept, analyses involving larger cohorts will be required to further investigate the clinical potential of HER2-HER3 dimerization status on CTCs.

## Data Availability

The data presented in this study are available on request from the corresponding author.

## Supplementary Materials

The following supporting information can be downloaded at: https://www.mdpi.com/article/10.3390/cancers14081890/s1, Table S1: mRNA primers, Table S2: Thermal Cycling Conditions for cDNA Amplification, Figure S1: Direct PLA signals detected on glass slides with MDA-MB (left) and 231 SK-BR-3 (right) cells in the pres- 444 ence of anti-HER2 and anti-HER3 primary antibodies—DAPI in blue, HER2-HER3 PLA signal in red-scale bar: 20 μm, Figure S2: Direct PLA signals detected on glass slides with SK-BR-3 cells in the presence of anti-HER2 and anti-HER3 453 primary antibodies (left) or only in presence of anti-HER3 primary antibody as negative control (right)—DAPI in blue, 454 HER2-HER3 PLA signal in red, Figure S3: Direct PLA signals detected on-chip with SK-BR-3 (left) and MDA-MB 231 (right) cells—DAPI in blue, HER2- 460 HER3 PLA signal in red, Figure S4: Comparison of PLA signals for SK-BR3 and MDA-MB 231 cell lines- N = 3 independent experiments. (*t*-test 464 analysis), Figure S5: mRNA expression for silencing of HER2, HER3 or both in SK-BR-3 cell, Figure S6: HER2 (right) or HER3 (left) gene silencing in SK-BR-3 cells. Both individual silencing induced a significant 476 decrease of PLA signals/cell, represented by the blue Gaussian curve shifted to the left. N = 3 independent experiments 477 for each condition, Figure S7: ALCINA- Circe PLA Inclusion chart.

## References

[1] Perou C.M., Sørlie T., Eisen M.B., van de Rijn M., Jeffrey S.S., Rees C.A., Pollack J.R., Ross D.T., Johnsen H., Akslen L.A. (2000). Molecular portraits of human breast tumours. Nature.

[2] Goutsouliak K., Veeraraghavan J., Sethunath V., De Angelis C., Osborne C.K., Rimawi M.F., Schiff R. (2019). Towards personalized treatment for early stage HER2-positive breast cancer. Nat. Rev. Clin. Oncol..

[3] Wolff A.C., Hammond M.E.H., Hicks D.G., Dowsett M., McShane L.M., Allison K.H., Allred D.C., Bartlett J.M., Bilous M., Fitzgibbons P. (2013). Recommendations for human epidermal growth factor receptor 2 testing in breast cancer: American Society of Clinical Oncology/College of American Pathologists clinical practice guideline update. J. Clin. Oncol..

[4] Yamashita-Kashima Y., Shu S., Yorozu K., Moriya Y., Harada N. (2017). Mode of action of pertuzumab in combination with trastuzumab plus docetaxel therapy in a HER2-positive breast cancer xenograft model. Oncol. Lett..

[5] Sawyers C.L. (2019). Herceptin: A fist assault on oncongenes that launched a revolution. Cell.

[6] Gala K., Chandarlapaty S. (2014). Molecular pathways: HER3 targeted therapy. Clin. Cancer Res..

[7] Zhao F., Huo X., Wang M., Liu Z., Zhao Y., Ren D., Xie Q., Liu Z., Li Z., Du F. (2021). Comparing Biomarkers for Predicting Pathological Responses to Neoadjuvant Therapy in HER2-Positive Breast Cancer: A Systematic Review and Meta-Analysis. Front. Oncol..

[8] Weitsman G., Barber P.R., Nguyen L.K., Lawler K., Patel G., Woodman N., Kelleher M.T., Pinder S.E., Rowley M., Ellis P.A. (2016). HER2-HER3 dimer quantification by FLIM-FRET predicts breast cancer metastatic relapse independently of HER2 IHC status. Oncotarget.

[9] Green A.R., Barros F.F., Abdel-Fatah T., Moseley P., Nolan C.C., Durham A.C., Rakha E.A., Chan S., Ellis I.O. (2014). HER2/HER3 heterodimers and p21 expression are capable of predicting adjuvant trastuzumab response in HER2+ breast cancer. Breast Cancer Res. Treat..

[10] Mattox A.K., Bettegowda C., Zhou S., Papadopoulos N., Kinzler K.W., Vogelstein B. (2019). Applications of liquid biopsies for cancer. Sci. Transl. Med..

[11] Heitzer E., Haque I.S., Roberts C.E.S., Speicher M.R. (2019). Current and future perspectives of liquid biopsies in genomics-driven oncology. Nat. Revm Genet..

[12] Eslami-S Z., Cortés-Hernández L.E., Cayrefourcq L., Alix-Panabières C. (2019). The different facets of liquid biopsy: A kaleidoscopic view. Cold Spring Harbor Persp. Med..

[13] Dianat-Moghadam H., Azizi M., Eslami-S Z., Cortés-Hernández L.E., Heidarifard M., Nouri M., Alix-Panabières C. (2020). The role of circulating tumor cells in the metastatic cascade: Biology, technical challenges, and clinical relevance. Cancers.

[14] Kloten V., Lampignano R., Krahn T., Schlange T. (2019). Circulating Tumor Cell PD-L1 Expression as Biomarker for Therapeutic Efficacy of Immune Checkpoint Inhibition in NSCLC. Cells.

[15] Vasseur A., Kiavue N., Bidard F.C., Pierga J.Y., Cabel L. (2021). Clinical utility of circulating tumor cells: An update. Mol. Oncol..

[16] Bidard F.C., Proudhon C., Pierga J.Y. (2016). Circulating tumor cells in breast cancer. Mol. Oncol..

[17] Saias L., Autebert J., Malaquin L., Viovy J.L. (2011). Desig, modeling and characterization of microfluidic architecture for high flow rate, small footprint microfluidic systems. Lab Chip.

[18] Autebert J., Coudert B., Champ J., Saias L., Tulukcuoglu Guneri E., Lebofsky R., Bidard F.C., Pierga J.Y., Farace F., Descroix S. (2015). High Purity Microfluidic Sorting and Analysis of Circulating Tumor Cells: Towards Routine Mutations Detection. Lab Chip.

[19] Delapierre F.-D., Mottet G., Taniga V., Boisselier J., Viovy J., Malaquin L. (2017). High Throughput Micropatterning of Interspersed Cell Arrays Using Capillary Assembly. Biofabrication.

[20] Kraus T., Malaquin L., Schmid H., Riess W., Spencer N.D., Wolf H. (2007). Nanoparticle Printing with Single-Particle Resolution. Nat. Nanotechnol..

[21] Söderberg O., Leuchowius K.J., Gullberg M., Jarvius M., Weibrecht I., Larsson L.G., Landegren U. (2008). Characterizing proteins and their interactions in cells and tissues using the in situ proximity ligation assay. Methods.

[22] Fredriksson S., Gullberg M., Jarvius J., Olsson C., Pietras K., Gústafsdóttir S.M., Östman A., Landegren U. (2002). Protein detection using proximity-dependent DNA ligation assays. Nat. Biotechnol..

[23] Subik K., Lee J.F., Baxter L., Strzepek T., Costello D., Crowley P., Xing L., Hung M.C., Bonfiglio T., Hicks D.G. (2010). The Expression Patterns of ER, PR, HER2, CK5/6, EGFR, Ki-67 and AR by Immunohistochemical Analysis in Breast Cancer Cell Lines. Breast Cancer.

[24] Miserere S., Mottet G., Taniga V., Descroix S., Viovy J.-L., Malaquin L. (2012). Fabrication of Thermoplastics Chips through Lamination Based Techniques. Lab Chip.

[25] Kujur P.K., Flores B.C.T., Ramalingam N., Chinen L.T.D., Jeffrey S.S. (2020). Advances in the Characterization of Circulating Tumor Cells in Metastatic Breast Cancer: Single Cell Analyses and Interactions, and Patient-Derived Models for Drug Testing. Adv. Exp. Med. Biol..

[26] Gómez-Sjöberg R., Leyrat A.A., Pirone D.M., Chen C.S., Quake S.R. (2007). Versatile, fully automated, microfluidic cell culture system. Anal. Chem..

[27] Wu Y.H., Lai M.Z. (2016). Measuring NLR Oligomerization V: In Situ Proximity Ligation Assay. Methods Mol. Biol..

