# Supplementary material for: Deciphering HER2-HER3 Dimerization at the Single CTC Level: A Microfluidic Approach
Source: Cancers (Basel). 2022 Apr 8;14(8):1890. doi: 10.3390/cancers14081890 (PMC9026778; doi:10.3390/cancers14081890)
Supplement: Supplementary file 1 [file cancers-14-01890-s001.zip › cancers-1659584-supplementary.pdf]

Table S1 : mRNA primers

Oligo Name	Sequence
human GAPDH forward	ACAGTTGCCATGTAGACC
human GAPDH reverse	TTGAGCACAGGGTACTTTA
human actin beta forward	ATATGAGATGCGTTGTTA
human actin beta reverse	AGTATTAAGGCGAAGATTA
human HER3 forward	CATCTCATCTCAGGAAGTG
human HER3 reverse	TCGGATAAGGAGTGAAGA
human HER2 forward	CTGTCCTGTTCACCACTCT
human HER2 reverse	TTCATCCTCATCATCTTCACATTG

Table S2 : Thermal Cycling Conditions for cDNA Amplification.

	PCR Stage	Temperature	Time
**Stage 1**	Hold	95°C	10min
**Stage 2**	Cycle (40 cycles)		
	Denature	95°C	15s
	Anneal/Extend	60°C	60s
**Stage 3**	Melt curve (Dissociation stage)	95°C	10s
		0.2°C Increments	
		60°C to 95°C	10s

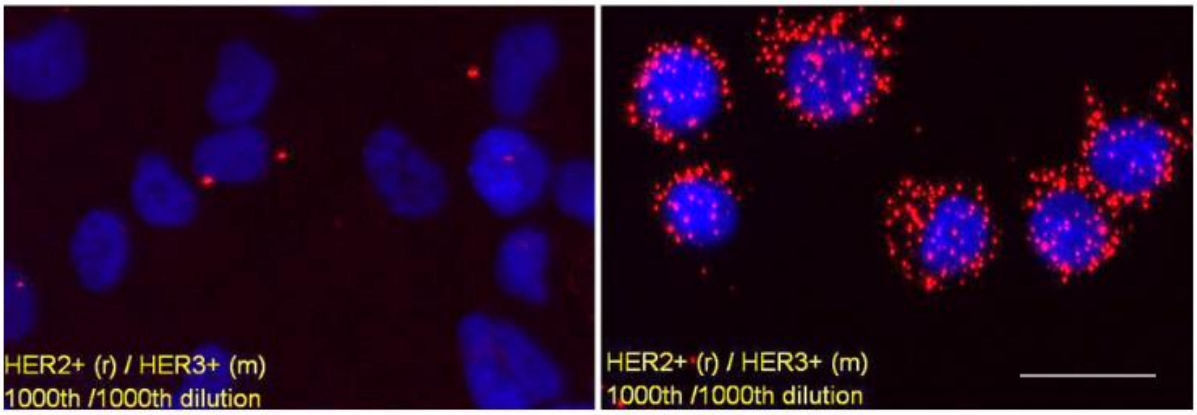

Figure S1: Direct PLA signals detected on glass slides with MDA-MB (left) and 231 SK-BR-3 (right) cells in the pres- 444 ence of anti-HER2 and anti-HER3 primary antibodies - DAPI in blue, HER2-HER3 PLA signal in red- scale bar : 20µm.

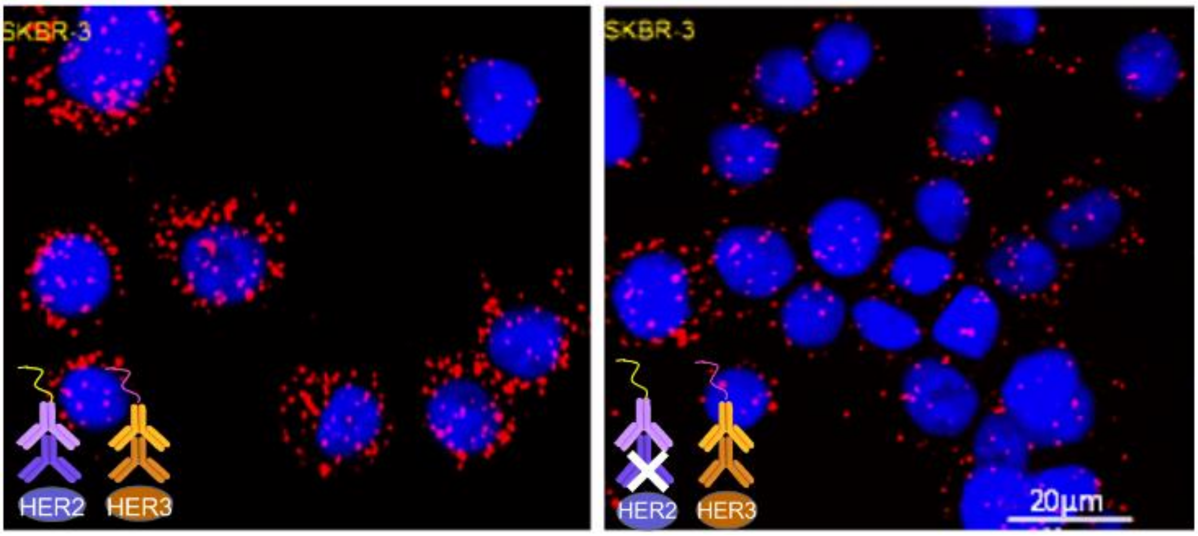

Figure S2: Direct PLA signals detected on glass slides with SK-BR-3 cells in the presence of anti-HER2 and anti-HER3 primary antibodies (left) or only in presence of anti-HER3 primary antibody as negative control (right)- DAPI in blue, HER2-HER3 PLA signal in red.

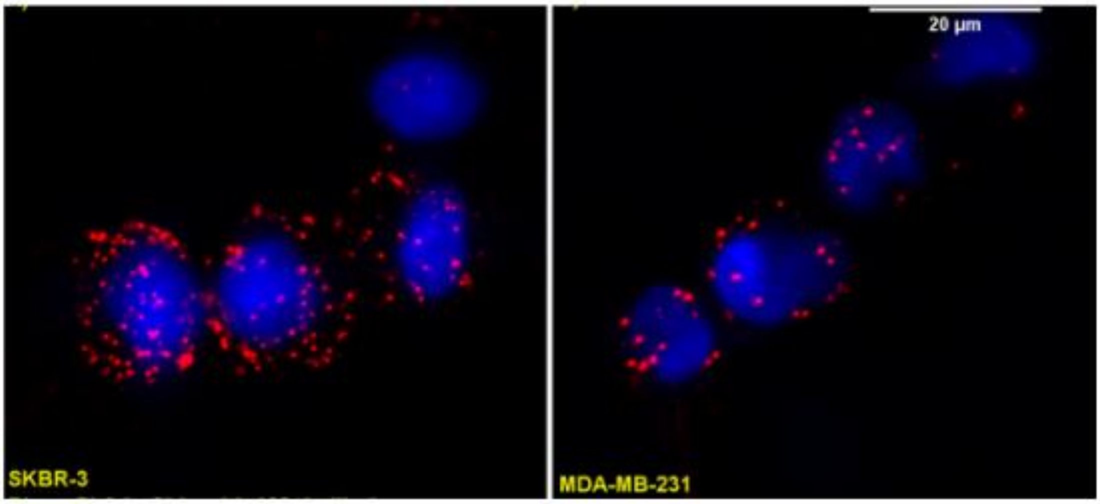

Figure S3: Direct PLA signals detected on-chip with SK-BR-3 (left) and MDA-MB 231 (right) cells - DAPI in blue, HER2- HER3 PLA signal in red.

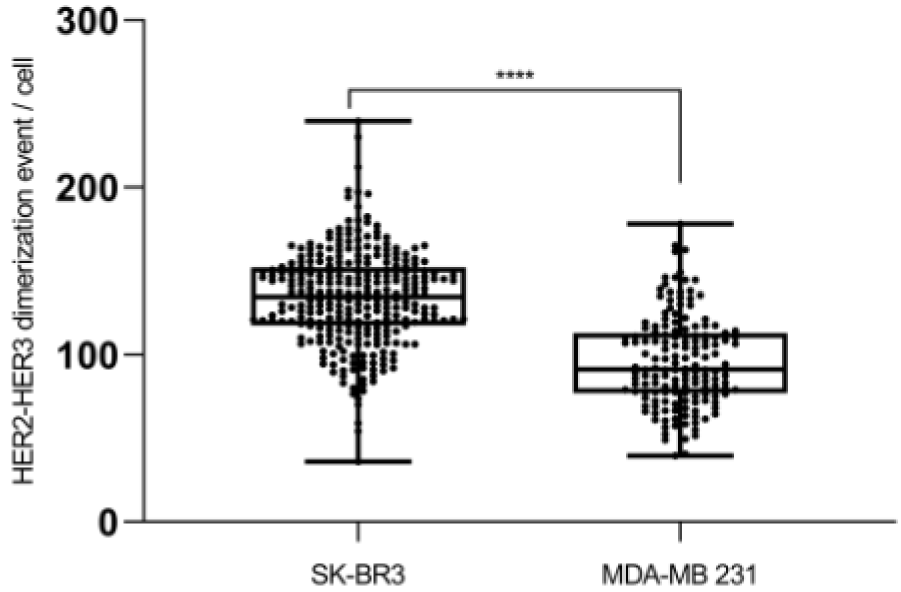

Figure S4: Comparison of PLA signals for SK-BR3 and MDA-MB 231 cell lines- N= 3 independent experiments. (t-test 464 analysis) **** $p < 0.0001$.

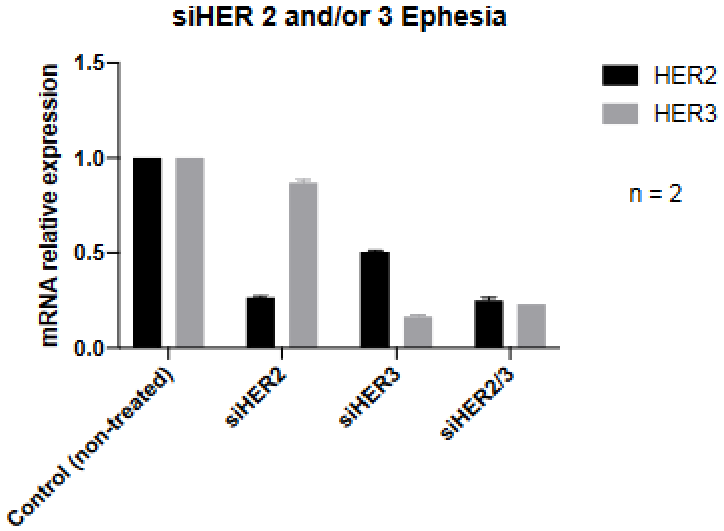

Figure S5 : mRNA expression for silencing of HER2, HER3 or both in SK-BR-3 cell.

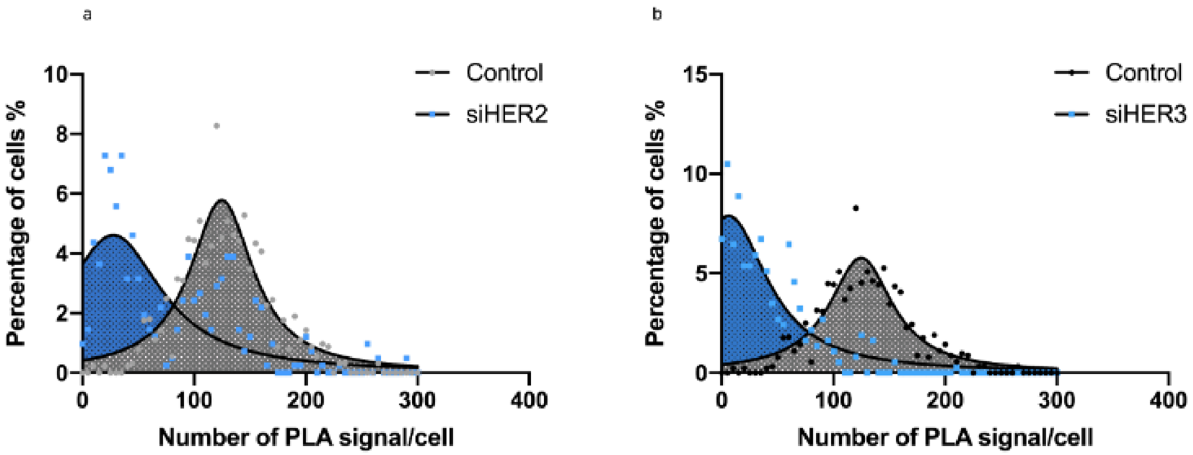

Figure S6: HER2 (right) or HER3 (left) gene silencing in SK-BR-3 cells. Both individual silencing induced a significant 476 decrease of PLA signals/cell, represented by the blue Gaussian curve shifted to the left. N= 3 independent experiments 477 for each condition.

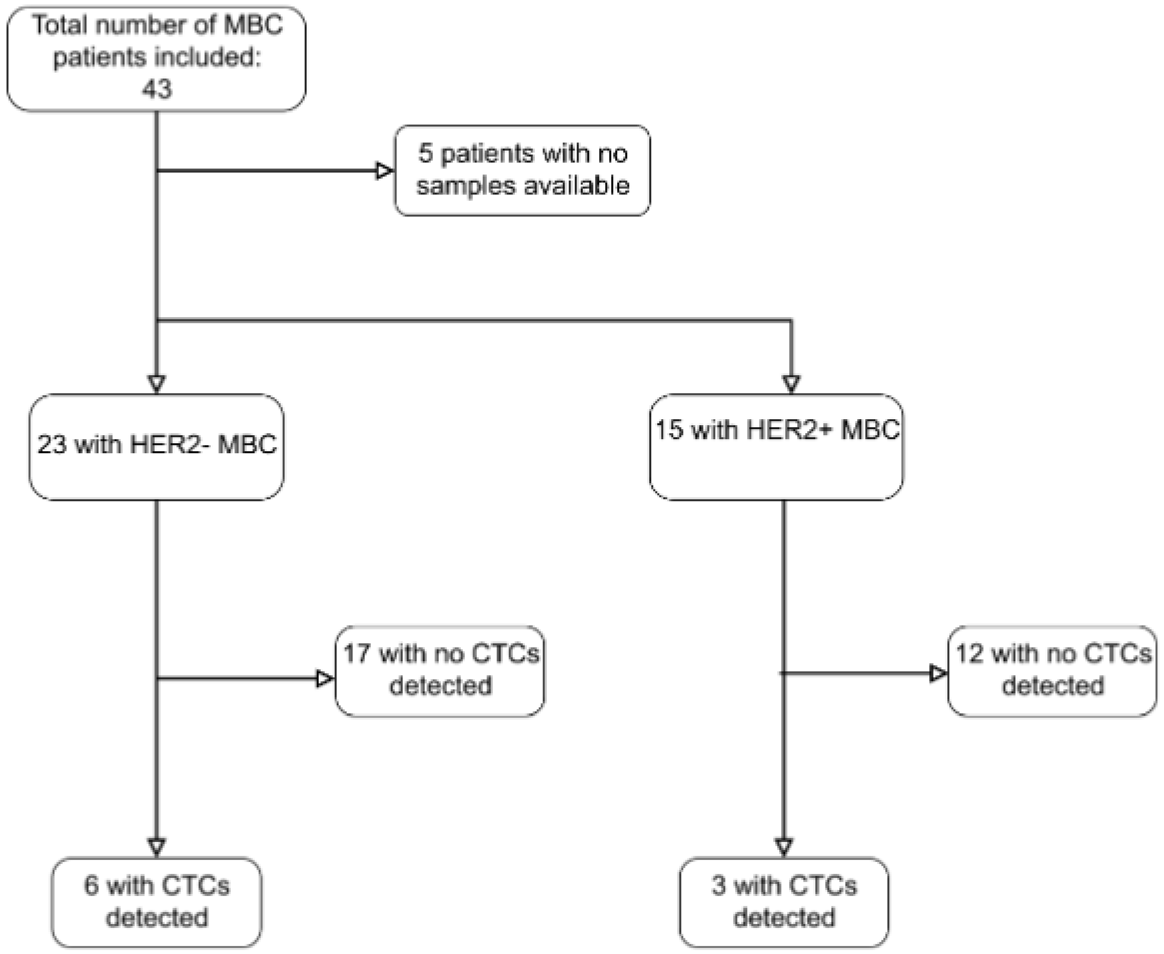

Figure S7 : ALCINA- Circe PLA Inclusion chart
